# Long term biventricular support with Berlin Heart Excor in a Septuagenarian with giant-cell myocarditis

**DOI:** 10.1186/s13019-015-0218-9

**Published:** 2015-01-31

**Authors:** Christian Bireta, Theodor Tirilomis, Marius Grossmann, Bernhard Unsöld, Rolf Wachter, Thorsten Perl, Ahmad Fawad Jebran, Friedrich Albert Schoendube, Aron Frederik Popov

**Affiliations:** 1 Department of Thoracic and Cardiovascular Surgery, University of Goettingen, Goettingen, Robert-Koch-Strasse 40, 37075 Goettingen, Germany; 2 Department of Cardiology and Pneumology, University of Goettingen, Robert-Koch-Strasse, 40, 37075 Goettingen, Germany; 3 Department of Anesthesiology, Emergency and Intensive Care Medicine, University of Goettingen, Robert-Koch-Strasse 40, 37075 Goettingen, Germany

## Abstract

Giant-cell myocarditis (GCM) is known as a rare, rapidly progressive, and frequently fatal myocardial disease in young and middle-aged adults. We report about a 76 year old male patient who underwent implantation with a biventricular Berlin Heart Excor system at the age of 74 due to acute biventricular heart failure caused by giant-cell myocarditis. The implantation was without any surgical problems; however, a difficulty was the immunosuppressive therapy after implantation. Meanwhile the patient is 76 years old and lives with circulatory support for about 3 years without major adverse events. Also, in terms of mobility in old age there are no major limitations. It seems that in even selected elderly patients an implantation of a long term support with the biventricular Berlin Heart Excor is a useful therapeutic option with an acceptable outcome.

## Background

Giant-cell myocarditis (GCM) is known as a rare, rapidly progressive, and frequently fatal myocardial disease in young and middle-aged adults. However, few cases have been reported on GCM in older patients. The paucity of reported cases on the elderly may reflect less frequent diagnosis or more or less fulminant course of disease in this population. Infection, autoimmune processes, and genetics have all been implicated in the pathogenesis of this disease, but the etiology is likely to be a complex multifactorial process. It is attributed to a T lymphocyte-mediated inflammation of the heart muscle and associates with systemic autoimmune diseases in 20% of cases [1,2]. The most common early manifestations are heart failure, ventricular arrhythmias, and atrioventricular block, but GCM may also appear as an acute myocardial infarction and rarely presents as an unexpected sudden cardiac death. Due to this unspecific clinical presentation of the patients, which may also be caused by other heart disease, the diagnosis of GCM fully depends on microscopy of the heart muscle with a sensitivity of 80% to 85% [1,2]. The histological hallmark of GCM is a multifocal inflammatory infiltrate manifested by many multinucleated giant cells and by extensive myocardial cell necrosis in the absence of granuloma formation [3]. Because of possible life threatening complications associated with GCM and the potential for benefit from treatment, early biopsy is recommended. An early diagnosis of GCM is crucial. Also, apart from standard heart failure therapy and physical rest a tailored immunosuppressive treatment may significantly alter the clinical course of the patients. The prognosis of patients with GCM is poor, and the probability of death or transplantation at 1 year from onset of symptoms is high [1,2]. Even after transplantation, there is a 20-25% GCM recurrence in the transplanted heart [4].

## Case presentation

A 74 year-old man was transferred to our institution with biventricular heart failure and suspected myocarditis. One week earlier, he was admitted to a local hospital due to decreased exercise tolerance and a rapidly worsening shortness of breath. Three weeks before, he had flu-like syndrome. Due to coronary heart disease the patient had undergone a coronary artery bypass grafting 20 years prior to the current admission. A recently performed angiogram showed that all bypass-grafts were competent.

On admission, his physical examination was unremarkable except for tachycardia and dyspnea. ECG revealed a sinus rhythm with a heart rate of approximately 120 bpm without significant repolarisation disturbances. Transthoracic echocardiography showed an increased left ventricular (LV) and-diastolic dimension of 58 mm with global thickening and severely reduced systolic function (ejection fraction 15%). No new regional wall abnormalities were noted. Chest X-ray showed a cardiomegaly and pleural effusion on the left side. Blood chemistry showed a slightly increased troponin T and creatine kinase isoenzyme MB. NT-pro brain natriuretic peptide (BNP) level was 18186 ng/L. Serologic tests for cardiotropic viruses were negative. The patient was transferred to the cardiac intensive care unit, and standard heart failure therapy was started including beta-blocker, diuretics, and angiotensin converting enzyme. Four days later, the patient had a clinical deterioration with worsening of dyspnea and severe hypotension and was stabilized by inotropic support including levosimendan therapy. Because of developing hemodynamic instability one day later with signs of pulmonary edema and catecholamine refractory cardiogenic shock a femoral veno-arterial extracorporeal membrane oxygenation (ECMO) was initiated to stabilize the patient and as bridge to decision. On the next day, endomyocardial biopsies were taken from the right ventricle (septum). Histological study revealed acute GCM with extensive myocardial cell necrosis and inflammatory cell infiltrates. Due to the poor prognosis of GCM, no improvement in biventricular function and no option for cardiac transplantation a biventricular Berlin Heart Excor system (BIVAD) was implanted on day 5 via ECMO support. In addition, the patient was started on double immunosuppressive therapy that included prednisolone and cyclosporine. Due to an infection of the left ventricular cannula (Staphylococcus aureus and Pseudomonas aeruginosa) the immunosuppressive therapy was stopped two month after implantation. The infection was successfully treated with antibiotics according the microbiologists recommendation. After a hospital stay of three months the patient is meanwhile 76 years old and lives with circulatory support for about 3 years with no problems and without major adverse events. The latest echocardiography showed still a biventricular failure. In terms of mobility in old age there are no major limitations.

## Discussion

Giant-cell myocarditis is known as a rare, rapidly progressive, and frequently fatal myocardial disease in young and middle-aged adults. However, few cases have been reported in older patients. GCM has multiple causes and the etiology is likely to be a complex multifactorial process which is attributed to T lymphocyte-mediated inflammation of myocardium. The prognosis of patients with GCM is poor and the probability of death or transplantation is high. Apart from standard heart failure therapy a tailored immunosuppressive treatment may significantly alter the clinical course of the patients [1,2]. A recent work from Kandolin et al. shows that combined immunosuppression may lead to a partial remission characterized by freedom from severe heart failure and better estimated transplant-free survival [5]. Mechanical circulatory support with intra-aortic balloon pump, ECMO, and ventricular assist device (VAD) therapy have all been used in GCM patients as bridge to transplantation or occasional recovery [1,6]. As in our case transplantation was not possible due to the high age of the patient. The only therapeutic option was implantation of a BIVAD. Critical circulatory status, previous cardiac operations, older age and associated multimorbidity are the key determinants rendering the conditions of permanent mechanical circulatory support (MCS) in patients of advanced age and are associated with a high postoperative mortality in this cohort [7]. Optimal patient selection and timing of MCS implantation are closely related to ethical issues. There is still a question based on which criteria can we predict the clinical course and when are we allowed to decide about the restriction of access to MCS in severely ill patients with limited prognosis. In our opinion, as well as by others, because of the high early mortality, the inclusion of patients with the combination of advanced age and profound ECMO implantation and all measurable values (e.g. creatinine, urea liver enzymes) were within normal range. He did not require dialysis and had no need for re-exploration for bleeding. And thirdly, he was neurologically alert responsive straight after ECMO implantation and expressed the wish for a BIVAD implantation after diagnosis assurance. We choose the Berlin Heart Excor system for some reasons. There is a huge experience with this system worldwide even in elderly patients. Also, long-term surviving patients with the Berlin Heart Excor required exceptionally few hospital readmissions resulting in a long out-of-hospital VAD support time. And finally the EXCOR® Adult is intended for use in acute or chronic ventricular failure refractory to optimal medical and interventional therapy especially for biventricular heart failure. Our patient is meanwhile 76 years old and lives with circulatory support for about 3 years with no major problems and without major adverse events.

## Conclusions

Even in selected elderly patients an implantation and long term support with the biventricular Berlin Heart Excor may useful as therapeutic option with an acceptable outcome.

## Consent

Written informed consent was obtained from the patient for publication of this Case report. A copy of the written consent is available for review by the Editor-in-Chief of this journal.

## Abbreviations

GCM: Giant-cell myocarditis
ECG: Electrocardiography
bpm: Beets per minute
LV: Left ventricular
BIVAD: Biventricular ventricular assist device
ECMO: Extracorporeal membrane oxygenation
MCS: Mechanical circulatory support
